# O-GlcNAc participates in the meiosis of aging oocytes by mediating mitochondrial function

**DOI:** 10.1530/REP-24-0138

**Published:** 2024-11-14

**Authors:** Chuwei Li, Zhang Qian, Hong Zhang, Xie Ge, Li Chen, Mengqi Xue, Ting Tang, Zhaowanyue He, Lu Zheng, Chun Cao, Kemei Zhang, Rujun Ma, Bing Yao

**Affiliations:** 1 Department of Reproductive Medicine, Jinling Hospital, Affiliated Hospital of Medical School, Nanjing University, Nanjing, Jiangsu, China; 2 Center of Reproductive Medicine, Jinling Hospital, Nanjing Medical University, Nanjing, China; 3 Department of Urology, Nanfang Hospital, Southern Medical University, Guangzhou, Guangdong, China

## Abstract

**In brief:**

O-GlcNAc plays an important role in many age-related diseases. This study shows that O-GlcNAc participates in oocyte aging and that reducing O-GlcNAc levels in aging oocytes improves oocyte quality.

**Abstract:**

With an increase in the mean age at parturition worldwide, female reproductive aging has become a key health problem. Advanced maternal age is reflected by decreased oocyte quality; however, the molecular mechanisms of oocyte aging are uncharacterized. O-linked N-acetylglucosamine (O-GlcNAc), a dynamic posttranslational modification, plays a critical role in the development of many age-related diseases; yet, it remains unclear whether and how O-GlcNAc participates in oocyte aging. Here, we found that global O-GlcNAc was elevated in normal biological aging mice oocytes (9 months), which were characterized by meiotic maturation failure and impaired mitochondrial function. Specifically, O-GlcNAc targeted the mitochondrial fission protein dynamic-related protein 1 to mediate mitochondrial distribution in the process of aging. Using the O-GlcNAcase (OGA) pharmacological inhibitor Thiamet-G and *Oga* knockdown (*Oga*-KD) to mimic the age-related high O-GlcNAc in young oocytes from 6–8 week-old mice mimicked the phenotype of oocyte aging. Moreover, reducing O-GlcNAc levels in aging oocytes restored spindle organization to improve oocyte quality. Our results demonstrate that O-GlcNAc is a key regulator of meiotic maturation that participates in the progression of oocyte aging.

## Introduction

Childbearing age has been obviously delayed since the 1970s (Secomandi *et al.* 2022). However, female fertility starts to decrease in a woman’s early 30s (Broekmans *et al.* 2009) and significantly declines beginning at age 35 (Yamamoto *et al.* 2010, Eijkemans *et al.* 2014). Decreased follicle numbers and deteriorated oocyte quality are the most notable characteristics of oocyte aging (Humm & Sakas 2013). These qualities pertain to the capabilities of oocytes to mature, be fertilized, and develop into an embryo (Gilchrist *et al*. 2008). Oocyte aging is a multi-factorial and heterogeneous process. Spindle assembly abnormality, oxidative stress, mitochondrial dysfunction and apoptotic are related to the deterioration of aging oocytes (Wakayama *et al.* 2004, Ma *et al.* 2005, Wang & Sun 2007, Zhang *et al.* 2019). As a consequence of age-related aberrations, the incidences of infertility, miscarriage, fetal mortality and birth defects have increased prominently in humans (Franasiak *et al.* 2014, Magnus *et al.* 2019). Thus, low-quality oocytes contribute to female fertility decline during aging. Therefore, investigating the underlying mechanisms of the process of oocyte aging is crucial to developing new strategies to improve the quality of aging oocytes.

O-linked-N-acetylglucosamylation (O-GlcNAcylation) is a widespread posttranslational modification (PTM) of nuclear, cytoplasmic and mitochondrial proteins that covalently attaches a single N-acetylglucosamine (GlcNAc) to serine and threonine residues in target proteins (Hart *et al.* 2007, Chatham *et al.* 2021). Only one pair of enzymes dynamically regulates the cycling of O-GlcNAcylation: O-GlcNAc transferase (OGT), which catalyzes the addition of O-GlcNAc moieties to proteins, and O-GlcNAcase (OGA), which removes the O-GlcNAc moieties from proteins (Alteen *et al.* 2021, Stephen *et al.* 2021). O-GlcNAcylation has been shown to be ubiquitous in nearly every aspect of cellular physiology, such as cell growth, division, metabolism and mitochondrial function (Bond & Hanover 2015). Notably, specific knockdown of *Oga* in mice oocytes impaired meiotic maturation *in vitro* (Lin *et al.* 2022). O-GlcNAc levels are increased in aging mouse and brown Norway rat tissues, including the brain, lungs, skin, thymus, testes and liver (Fülöp *et al.* 2008, Yang *et al.* 2012, Li *et al.* 2022), suggesting that O-GlcNAc may be involved in the process of aging. Importantly, many studies have shown that altered O-GlcNAc levels may be associated with many aging-related chronic diseases, including neurodegenerative disease, cancer, obesity and diabetes both in mice models and humans (Marotta *et al.* 2012, Jahangir *et al.* 2014, Banerjee *et al.* 2016, Lee *et al.* 2021). However, the O-GlcNAc levels of oocytes in aging and young mice and whether O-GlcNAc regulates female fertility remain unclear.

Mitochondria can be found in all eukaryotic cells and participate in many physical processes, such as energy supply, reactive oxygen species (ROS) production, the apoptosis cascade and calcium signaling (O’Sullivan *et al.* 2019). During oocyte growth, the number of mitochondria increases dramatically to meet the high demand for mitotic spindle formation, chromosome segregation and fertilization (Van Blerkom *et al.* 1995, Van Blerkom 2011, Eichenlaub-Ritter 2012). The embryo has no mitochondrial biogenesis ability until the blastocyst stage; oocyte-containing mitochondria are the only energy sources (Cummins 2004). Hence, mitochondria are the key indicators of oocyte quality (Tilly & Sinclair 2013), and mitochondrial dysfunction is considered to be related to oocyte aging (van der Reest *et al.* 2021). A previous study has shown that mitochondrial dysfunction leads to chromosomal nondisjunction and spindle assembly defects in aging oocytes (Guo & Yu 2019). Moreover, mounting evidence has shown that the cycling of O-GlcNAc can modulate mitochondrial function (Xue *et al.* 2022) and that excessive accumulation of O-GlcNAc impairs mitochondrial network homeostasis in diabetes (Zhao *et al.* 2016).

In our study, we used 9-month-old mice, which corresponded to late 30s in human (Boot *et al.* 1956, Zhang *et al.* 2019) models that displayed an obvious reproductive decline (Woods *et al.* 2017, Ziętek *et al.* 2021) to observe oocyte aging. Then, we utilized an OGA pharmacological inhibitor and *Oga* knockdown in young oocytes to imitate the excessive O-GlcNAc levels of aging oocytes. We found that increasing the O-GlcNAc level impairs oocyte meiotic maturation and that reducing the O-GlcNAc level in aging oocytes can restore oocyte quality. In addition, enhancing the O-GlcNAc level deteriorates the mitochondrial membrane potential (MMP) and results in abnormal mitochondrial distribution by reducing the expression level of phosphorylated dynamic-related protein 1 (DRP1) (Ser637), further leading to oxidative stress and early apoptosis. Our results highlight the role of O-GlcNAc in the aging of oocytes.

## Materials and methods

### Mice

Institute of Cancer Research (ICR) female mice were purchased from the Animal Core Facility of Nanjing Medical University. Young (6–8 weeks) and aged (9 months) ICR female mice were maintained under the same conditions at a constant temperature (20–23°C) with a humidity of 50–70% and a 12-h light/darkness cycle with free access to food and water. The mice were euthanized humanely by cervical dislocation to alleviate suffering during the collection of oocytes.

### Oocyte collection and *in vitro* maturation

To obtain fully grown germinal vesicle (GV) oocytes, ICR female mice were euthanized to puncture the ovaries. Oocytes were not hormonally stimulated prior to isolation. Cumulus cells were removed by repeated mouth pipetting under a stereomicroscope. Approximately 20–25 oocytes can be collected from one young mouse, and about 10–15 oocytes can be collected from one aged mouse. For *in vitro* maturation, GV oocytes were cultured in M16 medium (Sigma-Aldrich) under mineral oil at 37°C in an atmosphere of 5% CO_2_ for 8 or 14 h for different experiments, as described previously (Jin *et al.* 2022). Droplets of 50 µL of M16 with 30–40 oocytes were used for *in vitro* maturation. The experiment was repeated at least three times with 30–40 oocytes in the young, aged, TMG or O-GlcNActransferase inhibitor 1 (OSMI-1) groups.

### Thiamet-G and OSMI-1 treatment

Thiamet-G (APExBIO) (TMG), a water-soluble O-GlcNAcase inhibitor, was dissolved in phosphate-buffered saline (PBS) solution at 50 mM according to the manufacturer’s instructions. To increase the O-GlcNAc level, TMG was diluted in M16 medium to final concentrations of 10, 50 and 100 μM, as reported in previous studies (Zhou *et al.* 2019). The young oocyte group cultured in an M16 medium without inhibitors served as a negative control (0.2% PBS). The small molecule OGT inhibitor OSMI-1 (MedChemExpress), which was dissolved in DMSO to create a 60 mM stock solution, following previous studies in aging oocytes (Lee & Kwon 2020, Maucieri & Townson 2021) at concentrations of 10, 25 and 50 μM in M16 for 14 h. In the negative control group, oocytes were treated without inhibitors in the M16 medium (0.08% DMSO).

### siRNA knockdown

To knockdown *Oga*, microinjection of siRNA designed to specifically target *Oga* mRNA (Gene Pharma) was used. The *Oga* siRNA and NC-siRNA sequences are shown in Supplementary Table 1 (see section on supplementary materials given at the end of this article). GV stage oocytes were collected in an M2 medium with 1.5 μM milrinone (Sigma-Aldrich) to inhibit spontaneous germinal vesicle breakdown (GVBD). siRNA was diluted with water to give a stock concentration of 25 μM, then a 2.5 picolitre solution was injected into the fully-grown GV stage oocytes by Narishige microinjector. An equal concentration of negative control siRNA was also injected as a control.

### Western blotting

A pool of 90–100 oocytes per group was lysed in NuPAGE LDS sample buffer (4X) (Invitrogen Novex, Thermo Fisher Scientific) at 100°C for 5 min. The denatured protein extracts were first separated by SDS-PAGE and then transferred to PVDF membranes (Bio-RAD). The membranes were blocked in 5% BSA for 1 h and incubated overnight at 4°C with O-GlcNAc antibody RL2 (1:500) (Thermo Fisher Scientific), OGA antibody MEGA5 (1:500) (Proteintech), vinculin antibody (1:3000) (Proteintech), β-actin antibody (1:3000) (Proteintech) and GAPDH antibody (1:3000) (Proteintech). After multiple washes in TBST, the membranes were incubated with anti-mouse IgG, HRP–conjugated (1:5000) and anti-rabbit IgG, HRP–conjugated (1:5000) secondary antibodies (ImmunoWay Biotechnology Company) for 1 h at room temperature. After washing three times for 5 min, the protein bands were visualized using an ECL Plus Western Blotting Detection System (Tanon) and analyzed by ImageJ software. The intensity of O-GlcNAc bands was quantified in the whole lane. Protein intensities were normalized to reference proteins vinculin, β-actin and GAPDH in their respective lanes.

### Immunofluorescence and confocal microscopy

Oocytes were first fixed in 4% paraformaldehyde (PFA) for approximately 30 min at room temperature (RT) and then permeabilized with 0.5% Triton X-100 for 20 min. Afterward, the oocytes were blocked in 1% BSA-supplemented PBS for 1 h at RT and incubated with an anti-α-tubulin-with green fluorescein FITC antibody (1:200) (Sigma-Aldrich) and a rabbit polyclonal p-DRP1 (Ser637) antibody (1:50) (Affinity Bioscience) at 4°C overnight. Then, the oocytes were washed three times in wash buffer (0.1% Tween 20 and 0.01% Triton X-100 in PBS) and labeled with Alexa Fluor 488-conjugated antibodies (1:100) (ZSGB-BIO). Afterward, the oocytes were stained with Hoechst 33342 (20 μM) (Beyotime) for 15 min at RT. Finally, oocytes were observed with a laser scanning confocal microscope (Zeiss LSM 700 META) with the same scanning settings between biological and experimental repetitions. To ensure consistency in immunofluorescence measurements, the same reagents were utilized, and the staining duration and procedures were kept uniform. The fluorescence intensities were quantified using ImageJ software in the equatorial plane. The regions of interest (ROIs) were identified based on the oocytes’ morphology and the total fluorescence intensity within these areas was measured. Then the average intensity per unit area of the ROIs was calculated for comparison. Each experiment was repeated at least three times with about 35–45 oocytes.

### Assessments of MMP

The oocyte MMP (Δφm) was measured using an MMP assay kit (Beyotime C2003S). According to the instructions, oocytes were stained with 1 μL of JC-1 (200X) diluted in 199 μL of JC-1 staining buffer for 20 min at 37°C with 5% CO_2_. Oocytes were washed with Dulbecco's phosphate-buffered saline (DPBS) three times, for 3–5 min each time, and then moved to non-fluorescent glass slides. The fluorescence intensity of JC-1 monomers (red) and aggregates (green) was captured by confocal microscopy and analyzed using ImageJ software. The MMP was determined by measuring the ratio of red (JC-1 monomers) to green (JC-1 aggregates) fluorescence. Each experiment was repeated at least three times with about 35–45 oocytes.

### Detection of mitochondrial distribution

Oocytes were incubated in an M16 medium containing Mito-Tracker (0.1 μM) (Beyotime Biotechnology) for 30 min at 37°C and then washed three times with DPBS. After washing, the oocytes were counterstained with DAPI (20 μM) (Beyotime Biotechnology) to image the nucleus. The oocytes were mounted on nonfluorescent glass slides and observed under a laser scanning confocal microscope. The typical mitochondrial distribution manners were documented according to the method of a previous study (Miao *et al.* 2020). Each experiment was repeated at least three times with about 35–45 oocytes.

### Annexin V staining

Early apoptosis detection was performed by using an Annexin V-FITC Apoptosis Kit (Beyotime). To remove the zona pellucida, the viable oocytes were placed in Tyrode’s solution (Sigma-Aldrich) momentarily (about 30 s). Then, the oocytes were stained with 5 μL of Annexin V-FITC diluted in 195 μL of binding buffer for 15 min at RT in the dark. Afterward, the oocytes were washed three times in DPBS, placed on glass slides and observed under a laser scanning confocal microscope with the same scanning settings between biological and experimental repetitions. The fluorescence intensities were analyzed by ImageJ software. Each experiment was repeated at least three times with about 35–45 oocytes.

### Detection of intracellular ROS levels

The intracellular ROS level was measured with the fluorescent dye 2′,7′-dichlorofluorescein diacetate (DCFHDA) (KeyGEN bioTECH). Oocytes were incubated in an M2 medium with DCFHDA (10μM) for 30 min at 37°C in a 5% CO_2_ incubator. After three washes in DPBS, the oocytes of each group were observed by confocal microscope with the same scanning settings. The fluorescence intensity of M II stage oocytes was analyzed by ImageJ. Each experiment was repeated at least three times with about 35–45 oocytes.

### Statistical analysis

Statistical analyses were performed using SPSS software. The Shapiro–Wilk test was used to evaluate the normal distribution of the data before performing comparisons. Significant differences between groups were analyzed using Student’s *t* test and one-way ANOVA followed by multiple comparisons testing (Tukey *post hoc* test), and the data are presented as the mean ± s.d. *P* < 0.05 was considered to indicate statistical significance.

## Results

### Total O-GlcNAcylation increased in aging oocytes

**Figure 1:**
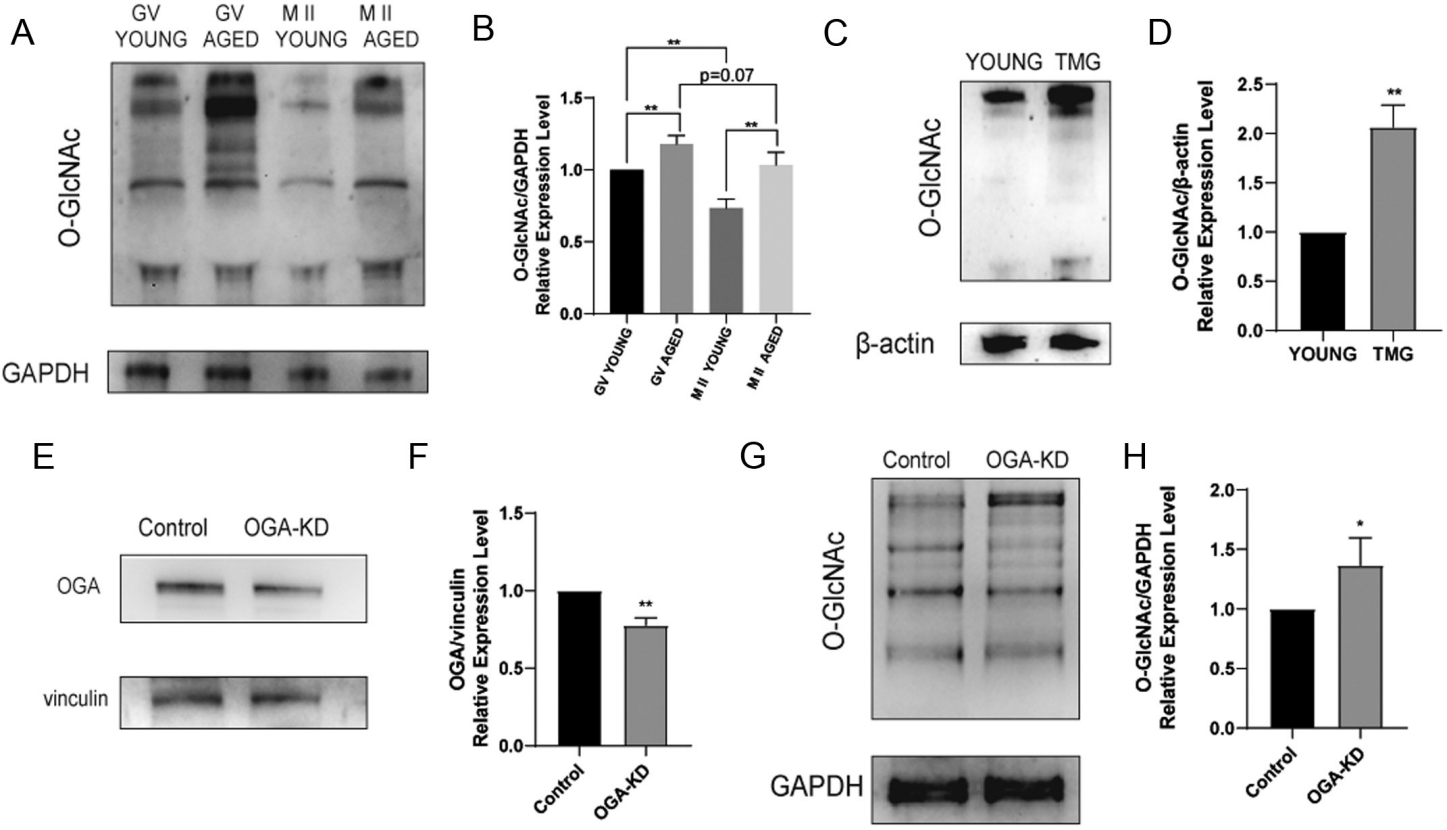
Total O-GlcNAc levels in the aging, young and TMG groups. (A) Western blot analysis of O-GlcNAc levels in protein extracts from GV and M II stage oocytes in the young (6–8 weeks) and aged (9 months). (B) Relative expression level of O-GlcNAc/GAPDH. (C) Western blot analysis of O-GlcNAc levels in protein extracts from denuded oocytes in young and TMG (young oocytes in a concentration of 50 μM TMG groups). (D) Relative expression level of O-GlcNAc/β-actin. (E) Western blot analysis of OGA level in protein extracts from denuded oocytes in control (young oocytes) and *Oga*-KD groups. (F) Relative expression level of OGA/vinculin. (G) Western blot analysis of O-GlcNAc level in protein extracts from denuded oocytes in control (young oocytes) and *Oga*-KD groups. (H) Relative expression level of O-GlcNAc/GAPDH. Each group consisted of approximately 90–100 oocytes for a single experiment. The data in (B), (D), (F) and (H) are presented as the mean ± s.d. from at least three independent experiments. *P < 0.05, **P < 0.01.

First, we investigated whether the O-GlcNAc modification exists in both young and aged oocytes during meiotic progression by western blot analysis. There was a sharp increase in total O-GlcNAcylation in both GV and metaphase II (M II) stage aged oocytes compared to young oocytes. Also, the O-GlcNAc levels decreased significantly from the GV stage to the M II stage in young oocytes. However, in the aged oocytes, there was only a declining trend (*P* = 0.07) (Fig. 1B and 1C). These findings indicate that the O-GlcNAc level is decreasing during meiotic maturation and is elevated in aging oocytes.

### Elevations in O-GlcNAc levels weakened meiotic maturation and disrupted spindle assembly

**Figure 2:**
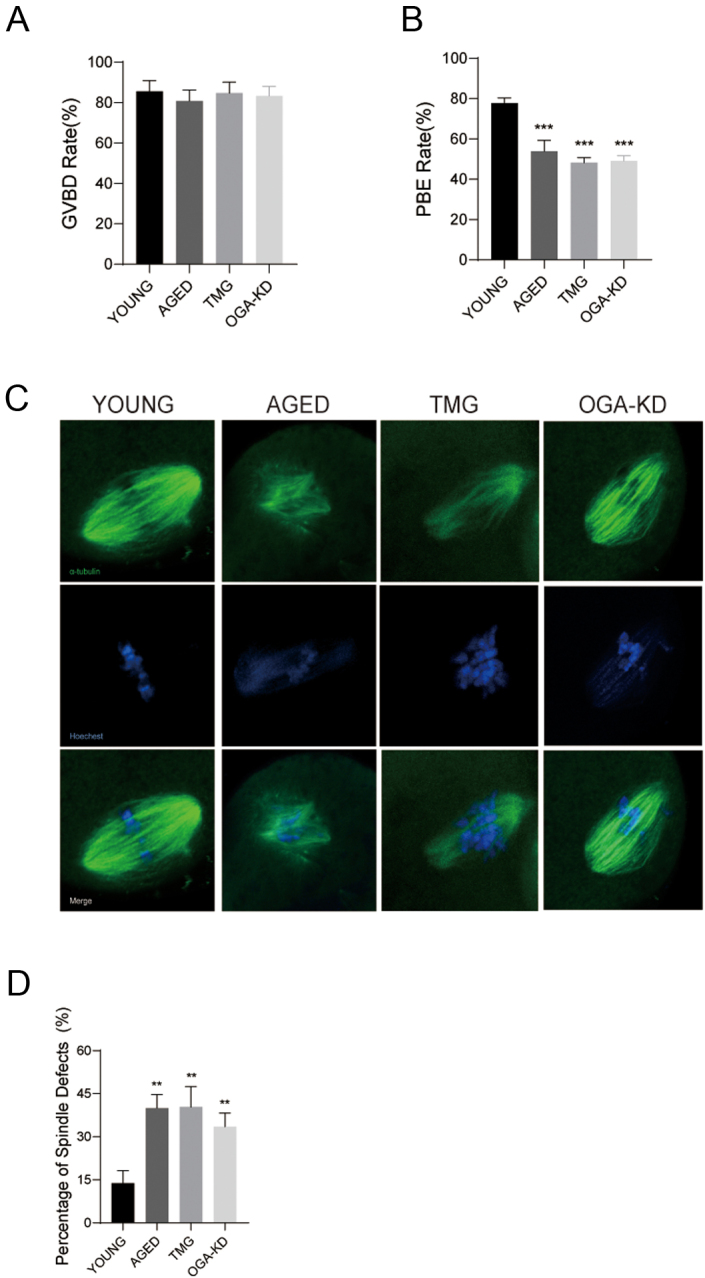
Effects of elevated O-GlcNAc on oocyte maturation and spindle assembly. (A) Rates of GVBD oocytes in the young (oocyte numbers = 135), aged (oocyte numbers = 105), TMG (oocyte numbers = 130) and *Oga*-KD groups (oocyte numbers = 105). (B) Rates of PBE oocytes in the young (oocyte numbers = 135), aged (oocyte numbers = 105), TMG (oocyte numbers = 130) and *Oga*-KD groups (oocyte numbers = 105). (C) Representative images of spindle morphologies in the young, aged, TMG and *Oga*-KD groups’ metaphase I (MI) and metaphase II (MII) oocytes. Spindle, green; chromosome, blue. Scale bar = 10 μm. (D) Rates of aberrant spindles in the young (oocyte numbers = 121), aged (oocyte numbers = 105), TMG (oocyte numbers = 120) and *Oga*-KD groups (oocyte numbers = 105). Each group consisted of approximately 35–45 oocytes for a single experiment. The data in (A), (B) and (D) are presented as the mean ± s.d. of at least three independent experiments. *P < 0.05, **P < 0.01, ***P < 0.001.

To investigate the effect of O-GlcNAc during oocyte aging, we added an inhibitor of OGA, TMG, to the M16 culture medium in young oocytes (called the TMG group in the text) to mimic an age-related O-GlcNAc increase *in vitro*. The first polar body extrusion (PBE) rate of 50 μM and 100 μM TMG groups was closest to the aging group (Supplementary Figure 1A and B). Therefore, we chose a dose of 50 μM for subsequent experiments. The effect of this dose on the O-GlcNAc level was verified by western blot (Fig. 1C). Also, we knocked down *Oga* in GV oocytes by siRNA. The effective depletion of *Oga* and the changed O-GlcNAc level were confirmed by western blot (Fig. 1D and E). To explore whether O-GlcNAcylation affects oocyte meiotic progression, GV oocytes isolated from young and aged mice were cultured *in vitro*. As shown in Fig. 2A, most of the oocytes in all groups reached the GVBD stage. However, the aged, TMG and *Oga*-KD groups displayed prominently decreased PBE rates (Fig. 2B), which indicated that elevated O-GlcNAcylation resulted in the meiotic arrest of young oocytes, analogous to the results in the aged group. It is generally acknowledged that meiotic failure is highly correlated with abnormal spindle assembly, so we next examined the subcellular spindle structure in metaphase II (MII)-stage oocytes by immunofluorescence. The majority of young oocytes exhibited a typical bipolar and barrel-shaped spindle with well-arranged chromosomes on the equatorial plate (Fig. 2C) and other dysmorphic spindles were classified as abnormal (Kilani & Chapman 2014, Tilia *et al.* 2016). In contrast, high frequencies of morphologically aberrant spindles were observed in the aged, TMG and *Oga*-KD groups (Fig. 2D). Given all these results, elevations in O-GlcNAcylation disrupted spindle assembly, thus deteriorating the maturation competence of oocytes. In view of the fact that TMG can achieve similar effects to *Oga*-KD, we used TMG for subsequent experiments.

### Elevated O-GlcNAcylation reduced MMP, impaired mitochondrial distribution and decreased the expression of p-DRP1 (Ser637)

**Figure 3:**
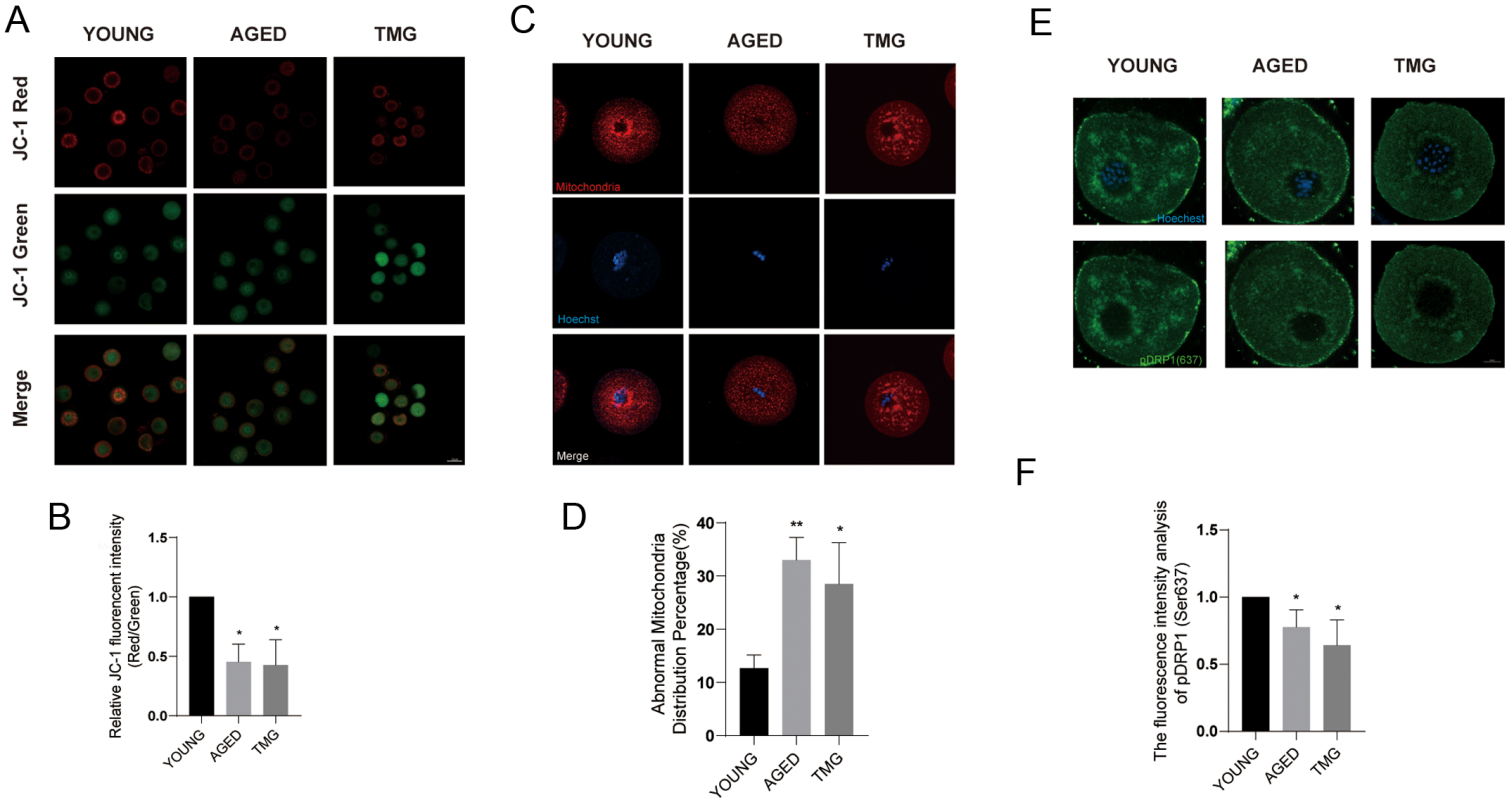
Effect of elevated O-GlcNAc on MMP, mitochondrial distribution and pDRP1(Ser637) levels in young, aged and TMG oocytes. (A) MMP was detected by JC-1 staining in the young, aged and TMG groups. Scale bar = 50 μm. (B) The ratio of red to green fluorescence intensity was calculated in the young (oocyte numbers = 112), aged (oocyte numbers = 107) and TMG groups (oocyte numbers = 120). (C) Representative images of mitochondrial distribution in the young, aged and TMG groups. Mitochondria, red; chromosome, blue. Scale bar = 25 μm. (D) Rates of abnormal mitochondrial distribution patterns in the young (oocyte numbers = 126), aged (oocyte numbers = 109) and TMG groups (oocyte numbers = 115). (E) Representative photographs of the immunofluorescence staining of p-DRP1 (Ser637) in the young, aged and TMG groups. p-DRP1 (Ser637), green; chromosome, blue. Scale bar = 10 μm. (F) Fluorescence intensity analysis of p-DRP1 (Ser637) in the young (oocyte numbers = 109), aged (oocyte numbers = 105) and TMG groups (oocyte numbers = 110). Each group consisted of approximately 35–45 oocytes for a single experiment. The data in (B), (D) and (F) are presented as the mean ± s.d. from at least three independent experiments. *P < 0.05.

Notably, the functional integrity of mitochondria is regarded as one of the most significant factors in oocyte meiosis. We assessed the MMP, which reflects mitochondrial function, by JC-1 staining. Mitochondria with high MMP represented red fluorescence, while mitochondria with low MMP represented green fluorescence (Fig. 3A). The red/green ratio was measured. As shown in Fig. 3B, the aged and TMG groups exhibited obviously lower MMP values than the young group. We then observed the distribution of mitochondria in MI-stage oocytes from the three groups by MitoTracker staining. The regular mitochondrial spreading pattern was characterized by aggregation in the periphery of the spindle (Fig. 3C). Aberrant positioning and mitochondrial signal weakening are considered abnormal. In the aged and TMG groups, the proportion of the normal distribution was decreased compared to the young group (Fig. 3D) Mitochondria change their distribution through constant fission and fusion, which rely on several important proteins. Drp1 is essential for mitochondrial fission and is activated by phosphorylation. One of the phosphorylated sites, Ser637, can also be modulated by O-GlcNAc, and there is a competitive relationship between the two PTMs (Gawlowski *et al.* 2012). Therefore, we examined the levels of p-DRP1 (Ser637) by immunofluorescence and found that in the aged and TMG groups, the levels of p-DRP1 (Ser637) were markedly decreased. Altogether, the results indicate that TMG may impair mitochondrial function by modulating the level of p-DRP1 (Ser637).

### Elevated O-GlcNAcylation leads to excessive ROS level and apoptosis

**Figure 4:**
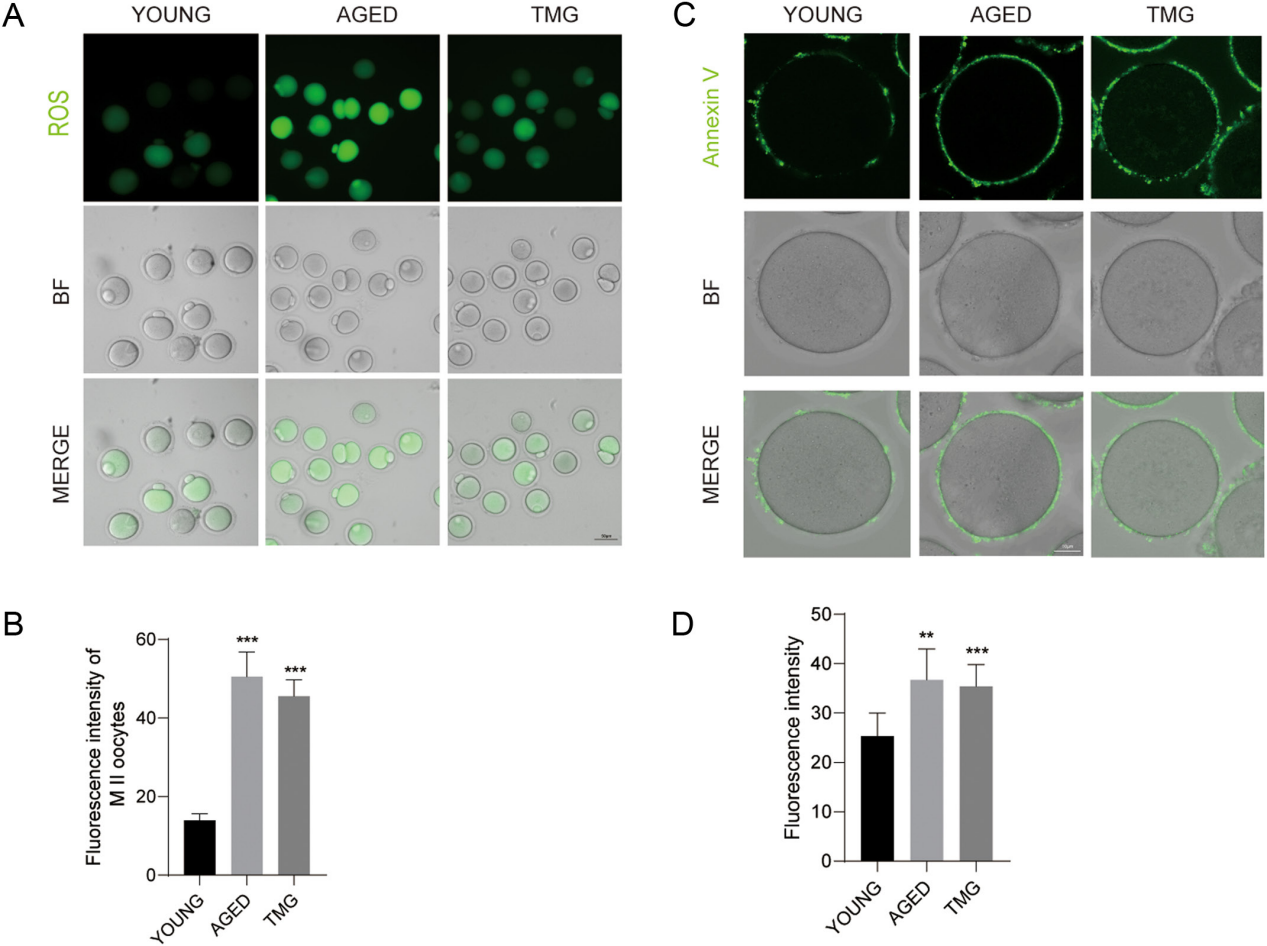
Effects of elevated O-GlcNAc on oxidative stress and early apoptosis. (A) Representative images showing the ROS levels in the young, aged and TMG groups. ROS, green. Scale bar = 50 μm. (B) The fluorescence intensities of M II stage oocytes’ ROS signals were measured in the young (oocyte numbers = 127), aged (oocyte numbers = 105) and TMG groups (oocyte numbers = 122). (C) Representative images of early apoptotic oocytes in the young, aged and TMG groups. Scale bar = 25 μm. (D) The fluorescence intensity of the Annexin V signals in the young (oocyte numbers = 130), aged (oocyte numbers = 106) and TMG groups (oocyte numbers = 112). Each group consisted of approximately 35–45 oocytes for a single experiment. The data in (B) and (D) are presented as the mean ± s.d. from at least three independent experiments. **P < 0.01, ***P < 0.01.

As mitochondrial dysfunction may cause oxidative stress, we examined the ROS levels in oocytes among the three groups. We found that much higher M II stage oocytes’ ROS signals appeared in the aged and TMG groups than in the young group (Fig. 4A and B). Also, mitochondrial lesions are usually associated with up-regulation of extrinsic apoptosis (Bastian *et al.* 2021, Jiang *et al.* 2023). Thus, we stained Annexin V in the young, aged and TMG groups. As expected, the aged and TMG groups showed a relatively stronger fluorescence intensity of the Annexin V signal than the young group in the oocyte membrane (Fig. 4C and D). Notably, we found that TMG led to excessive ROS and early apoptosis.

### Decreased levels of O-GlcNAc in aged oocytes can attenuate spindle defects

**Figure 5:**
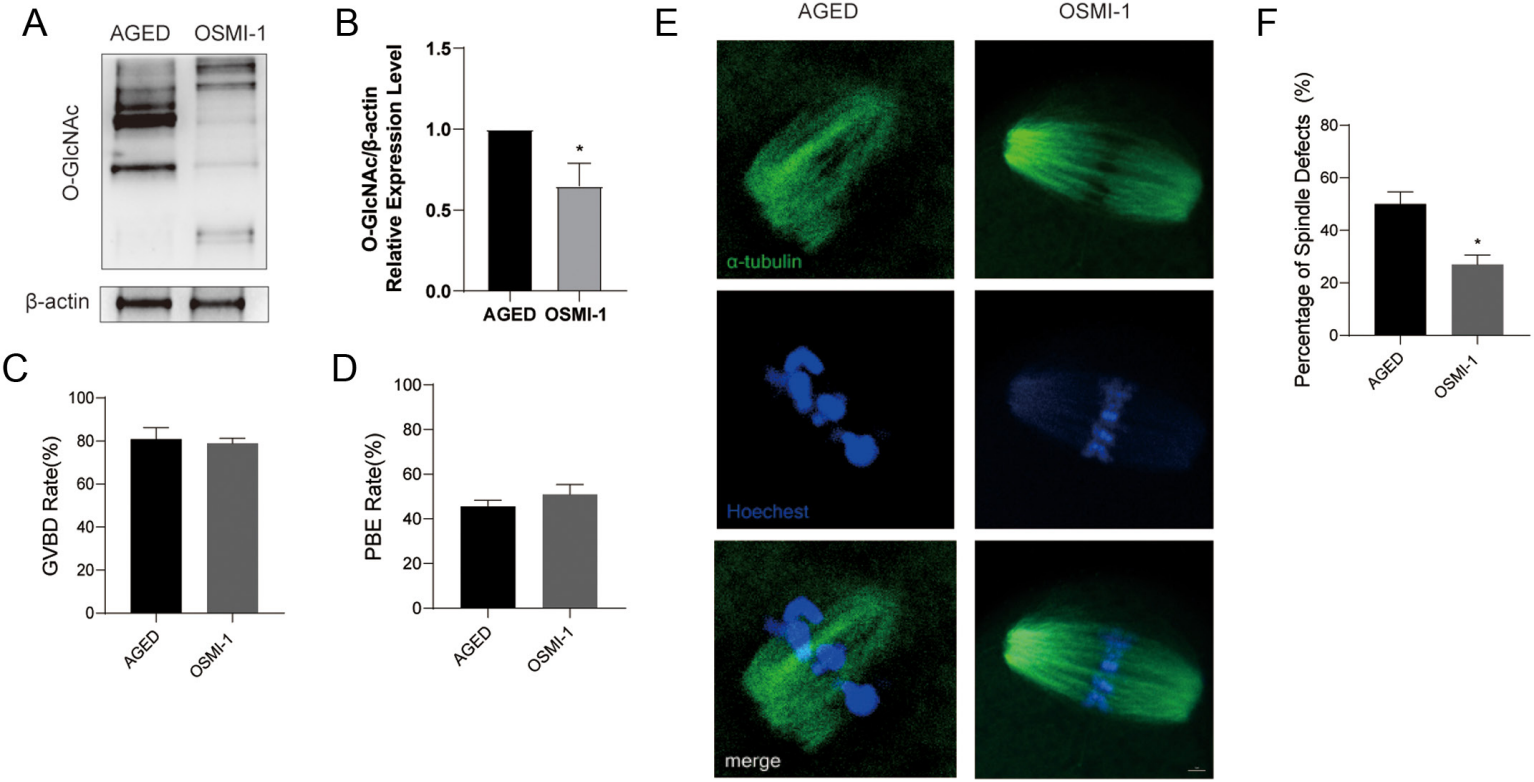
Effects of decreased O-GlcNAc on meiotic maturation and spindle assembly in aged oocytes. (A) Immunoblot analysis of O-GlcNAc levels in protein extracts from denuded oocytes in the aged and OSMI-1 (25 μM) groups. (B) Relative expression level of O-GlcNAc/β-actin. (C) Rates of GVBD of oocytes in the aged (oocyte numbers = 106) and OSMI-1 groups (oocyte numbers = 105). (D) Rates of PBE of oocytes in the aged (oocyte numbers = 106) and OSMI-1 groups (oocyte numbers = 105). (E) Representative pictures of spindle morphologies in young, aged and TMG oocytes. Spindle, green; chromosome, blue. Scale bar = 10 μm. (F) Rates of spindle defects in the aged (oocyte numbers = 108) and OSMI-1 groups (oocyte numbers = 105). Each group consisted of approximately 90–100 oocytes for a single immunoblot experiment. Each group consisted of approximately 35–45 oocytes for a single immunofluorescence experiment. The data in (B), (C), (D) and (F) are presented as the mean ± s.d. from at least three independent experiments. *P < 0.05.

Given our previous results, we next investigated whether decreasing O-GlcNAc levels in aging oocytes can restore developmental competence by adding the specific OGT inhibitor OSMI-1 to the M16 culture medium (called the OSMI-1 group in the text). We assessed the effects of different concentrations of OSMI-1 on GVBD and the first PBE rate in aging oocytes. The OSMI-1 had no effect on GVBD and PBE rate in aging oocytes (Supplementary Figure 3A and B). There was a decline in O-GlcNAc after treatment with the most effective concentration of 25 μM OSMI-1, as shown by western blotting (Fig. 5A). Hence, 25 μM was used for the following experiments (Fig. 5B, C, D and E). Interestingly, *in vitro* treatment of aging oocytes with OSMI-1 significantly reduced the percentage of spindle defects (Fig. 5D and E) and apoptosis (Supplementary Figure 2C and D). These data indicate that OSMI-1 can promote aging oocyte quality by reducing the level of O-GlcNAc.

## Discussion

In our study, we demonstrated that the levels of O-GlcNAc were elevated in both GV and M II stage aging mouse oocytes and that excessive O-GlcNAc impaired oocyte maturation by regulating mitochondrial function. In aging oocytes, we found a normal GVBD rate and a decreased PBE rate with defective spindle organization. Furthermore, our research indicated that there was abnormal mitochondrial distribution and reduced MMP in aged oocytes. We mimicked the age-related increase in O-GlcNAc in young oocytes and found that the young oocytes exhibited phenotypic changes characteristic of aging oocytes to a great extent. In addition, restoring O-GlcNAc homeostasis in aging oocytes significantly rescued the spindle structure. These findings may provide new insights for improving the quality of aged oocytes.

Previous studies have shown that O-GlcNAc participates in the meiosis of female and male gametes (Lefebvre *et al.* 2004, Dehennaut *et al.* 2008, Dehennaut *et al.* 2009, Frank *et al.* 2014, Zhou *et al.* 2019, Qian *et al.* 2023). Similar to a previous study, we found that during meiotic progression, the O-GlcNAc level decreased in oocytes (Lin *et al.* 2022). However, the basic O-GlcNAc level in aged GV stage oocytes was excessively high. Consequently, during the meiotic process, O-GlcNAc may be impeded from decreasing to a certain level. This hindrance could contribute to disruptions in meiotic maturation. In nonmammalian Xenopus oocytes, increases in O-GlcNAc levels after the addition of the O-GlcNAc substrate glucosamine impair maturation kinetics (Slawson *et al.* 2002). In our study, we used TMG instead of glucosamine because according to a previous study, glucosamine changes the O-GlcNAc level only in cumulus cells, not in oocytes (Sutton-McDowall *et al.* 2006). Consistent with a recent report (Lin *et al.* 2022), our data revealed that the disrupted O-GlcNAc level impaired the PBE rate, which is critical in oocyte maturation, but had no effect on the GVBD rate. PBE failure was accompanied by irregular chromosome separation, which was usually caused by defective spindle assembly. Therefore, we examined spindle morphology in aging oocytes and found an increased frequency of abnormal spindle formation. Similarly, aberrant O-GlcNAc due to *Oga* knockdown in mouse embryonic fibroblasts impairs cytokinesis and increases the occurrence of lagging chromosomes, which are mostly caused by spindle dysfunction (Yang *et al.* 2012). Additionally, mitotic faults and spindle disorganization occur in somatic cells after disruption of O-GlcNAc (Tan *et al.* 2013, Lanza *et al.* 2016). Our results are consistent with these findings, as a higher occurrence of spindle defects was detected in the TMG group. Considering the possible off-target effects of the inhibitor ((Yu *et al.* 2012)), we also performed the above experiments through the genetic depletion of *Oga* and got similar results. The findings indicate that disruption of O-GlcNAc impairs spindle assembly, inducing nuclear maturation failure.

Oocyte maturation includes nuclear maturation, which is marked as the first PBE, and cytoplasmic maturation (Conti & Franciosi 2018). Mitochondria play a critical role during oocyte maturation, and their redistribution is indispensable for cytoplasmic maturation (FitzHarris *et al.* 2007, Dalton & Carroll 2013). Our data revealed that the mitochondrial distribution was disrupted in aging and TMG oocytes. This result is consistent with a previous study showing that abnormal mitochondrial aggregates induced developmental retardation of oocytes (Nagai *et al.* 2006). Mitochondrial distribution relies on multiple fission and fusion protein components to form an interconnected network of mitochondrial clusters that can meet energy demands at different stages of oocyte development (da Silva *et al.* 2014, Wai & Langer 2016). Drp1 is essential for mitochondrial fission, and its active form is modulated by phosphorylation and O-GlcNAcylation (Cereghetti *et al.* 2008). This protein is phosphorylated at two sites, Ser616 and Ser637, and both sites can regulate mitochondrial fission (Chang & Blackstone 2007). As serine sites can also be O-GlcNAcylated, there is considerable crosstalk between phosphorylation and O-GlcNAc (van der Laarse *et al.* 2018). According to a previous study (Gawlowski *et al.* 2012), high O-GlcNAc levels decrease the levels of p-DRP1 (Ser637) and disrupt mitochondrial function. Therefore, we hypothesize that elevated O-GlcNAc may change the balance of mitochondrial fission proteins to regulate mitochondrial function. However, further investigation is needed.

Mitochondria are major sites of ROS production (Bentov & Casper 2013). However, mitochondria are susceptible to ROS-mediated damage (Keefe *et al.* 1995, Hou *et al.* 2019), which leads to mitochondrial dysfunction. This vicious cycle results in imbalanced ROS homeostasis in cells (Shigenaga *et al.* 1994). Moreover, increased ROS and apoptosis were observed in aging oocytes (Ma *et al.* 2005, Sasaki *et al.* 2019). In our study, ROS accumulation was elevated and the intensity of the Annexin V signal was increased in the aging group, consistent with previous studies showing that high O-GlcNAc levels were linked to increased apoptosis (Huang *et al.* 2011, Shi *et al.* 2015). However, the toxic effects of TMG cannot be ruled out, and particular cellular apoptosis pathways happening under high O-GlcNAc levels need further verification. After treatment with TMG, young oocytes displayed enhanced ROS levels and a high frequency of early apoptosis. According to some studies, high levels of O-GlcNAc may augment the degree of modification of several antioxidant enzymes, resulting in reductions in their protein content and decreases in total enzymatic activity (Arambašić *et al.* 2013, Dinić *et al.* 2013).

From the above results, we conclude that a high level of O-GlcNAc participates in oocyte aging, impeding meiotic progression by mediating mitochondrial function. This conclusion suggests that O-GlcNAc homeostasis is essential to cellular function. Because there is only one pair of enzymes that regulate the modification of O-GlcNAc and because an OGA-selective activator has not yet been established (Chatham *et al.* 2021), we chose the OGT inhibitor OSMI-1 to alleviate the excessive O-GlcNAc levels in aging oocytes. However, no statistically significant differences in PBE rates were observed between these groups and the aging group. We speculate that this might be due to the complexity of oocyte aging. Multiple molecular mechanisms are involved in oocyte aging (Secomandi *et al.* 2022). Therefore, only targeting O-GlcNAc may not improve the maturation rate significantly. However, OSMI-1 treatment prominently improved spindle assembly. This could have been due to the subcellular position of OGT. OGT colocalizes with spindles in mouse, bovine and human gametes (Slawson *et al.* 2005, Slawson & Duncan 2015, Zhou *et al.* 2019), and several spindle-associated proteins have been shown to contain O-GlcNAc sites (Slawson *et al.* 2005, Wang *et al.* 2010, Tan *et al.* 2013, Lanza *et al.* 2016, Tan *et al.* 2017). Therefore, we speculate that the OGT inhibitor improved spindle organization by changing the O-GlcNAc modification of several spindle proteins.

**Figure 6:**
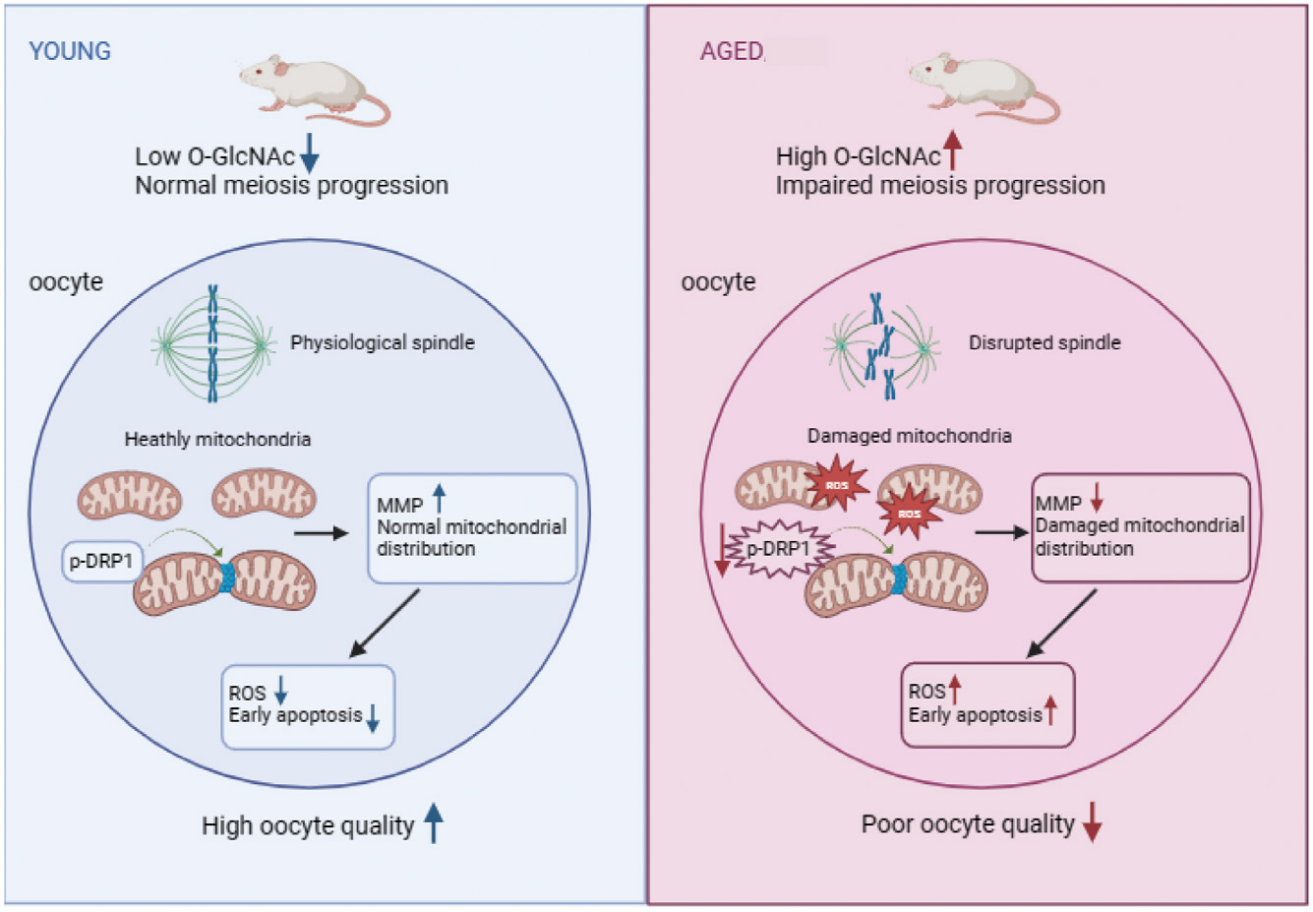
The mechanism by which O-GlcNAc participates in oocyte aging.

In conclusion, our results demonstrate that O-GlcNAc levels are increased in aging oocytes and that the increased O-GlcNAc participates in the progression of aging. Excessive O-GlcNAc induces meiotic maturation failure by mediating mitochondrial function. Furthermore, our data confirm that reducing O-GlcNAc levels restores spindle assembly in aging oocytes (Fig. 6). Overall, our work reveals that the PTM O-GlcNAc governs aging progression and provides a potential molecular target to improve aging oocyte quality.

## Supplementary Materials

Supplementary Material

## Declaration of interest

There is no conflict of interest that could be perceived as prejudicing the impartiality of the research reported.

## Funding

This work was supported by the National Natural Science Foundation of Chinahttp://dx.doi.org/10.13039/501100001809 (grant nos 82271687; U22A20277 and 81971373) and Jiangsu Provincial Medical Key Discipline Cultivation Unit (grant no. JSDW202215).

## Ethical Approval

Animal experiments were performed with permission from the Ethics Committee of Nanjing Jinling Hospital (2022DZGKJDWLS-00149) and conducted according to the NIH Guide for the Care and Use of Laboratory Animals.

## Author contribution statement

All authors listed have made a substantial, direct and intellectual contribution to the work, and approved it for publication. LCW, QZ and ZH conceived the study design, conducted the experiments, analyzed the data and wrote the manuscript. GX, CL and MRJ helped to design the experiments. XMQ, TT, HZWY, ZL, CC and ZKM participated in the experiments. MRJ and YB provided the funding. MRJ and YB reviewed the manuscript and provided substantial advice.
