# Supplementary material for: O-GlcNAc participates in the meiosis of aging oocytes by mediating mitochondrial function
Source: Reproduction. 2024 Nov 14;168(6):e240138. doi: 10.1530/REP-24-0138 (PMC11623119; doi:10.1530/REP-24-0138)
Supplement: Supplementary Material [file supplementary_material.pdf]

Table S1. siRNA sequences

Gene name	5'- sequence-3'
si-OGA-1	GGGACAUCAAGAGUAUAAUTT
si-OGA-2	GCCAAUUGAUGGAGCAAAUTT
si-OGA-3	GGUCCCUACAAAGGAAGAUTT
si-OGA-4	GACCAAUGGACACGGAUAATT
si-NC	UUCUCCGAACGUGUCACGUTT

Supplemental Figure 1

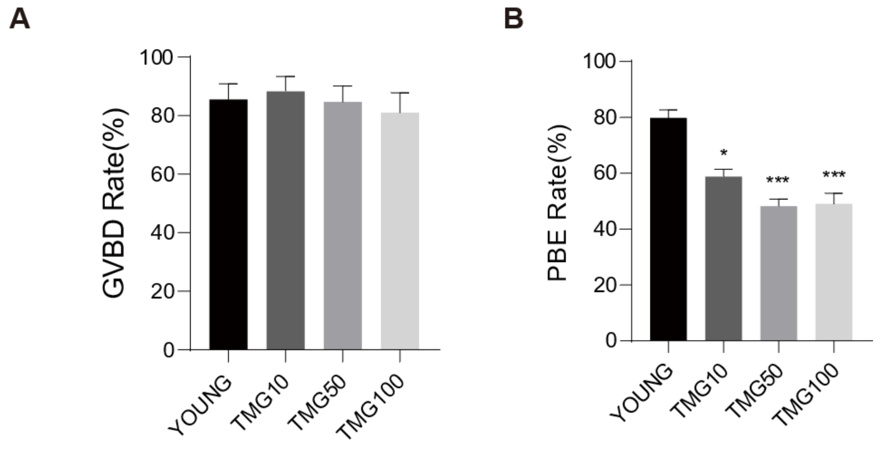

Figure S1. Effects of different doses of TMG on the in vivo maturation of young oocytes.

(A) Rates of GVBD of oocytes in the young and 10, 50 and 100μM TMG groups.

(B) Rates of PBE of oocytes in the young and 10, 50 and 100μM TMG groups.

Each group consisted of approximately 35-45 oocytes for a single experiment. The data in (A) and (B) are presented as the mean ± SD from at least three independent experiments. *$p < 0.05$, ***$p<0.01$.

Supplemental Figure 2

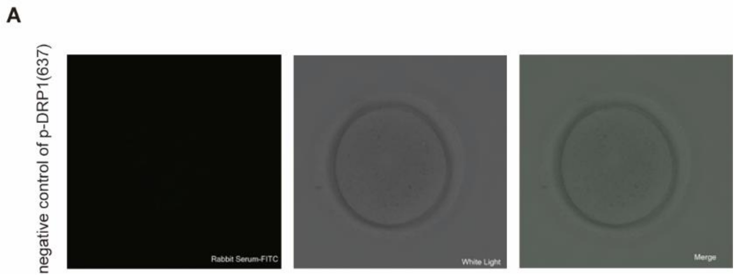

Figure S2. The negative control of p-DRP1(637).

(A) The image of negative control of p-DRP1(637) of oocyte.

Supplemental Figure 3

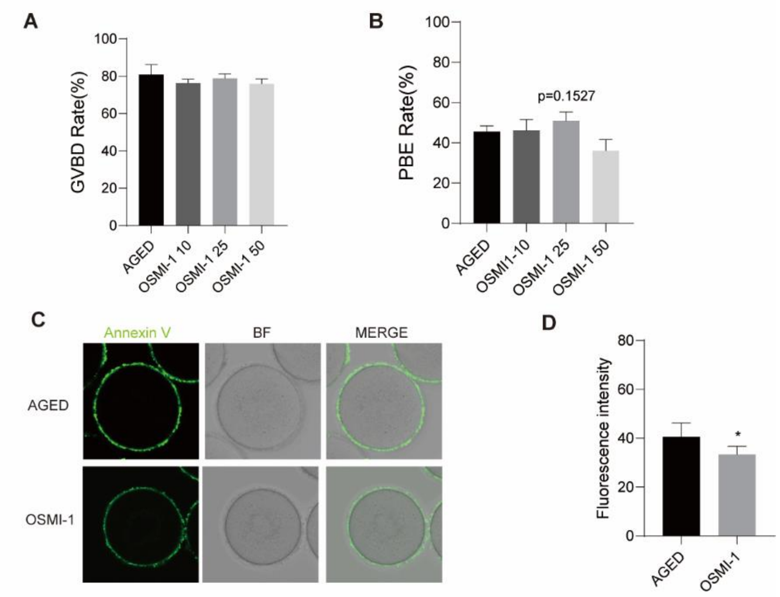

Figure S3. Effects of different doses of OSMI-1 on the in vivo maturation of aged oocytes.

(A) Rates of GVBD of oocytes in the young and 10, 25 and 50μM OSMI-1 groups.

(B) Rates of PBE of oocytes in the young and 10, 25 and 50μM OSMI-1 groups.

(C) The fluorescence intensity of Annexin V signals in the aged and OSMI-1 groups.

Each group consisted of approximately 35-45 oocytes for a single experiment. The data in (A), (B) and (C) are presented as the mean ± SD of at least three independent experiments. *$p < 0.05$.
